# Integrated Metabolomics and Proteomics Analysis of Urine in a Mouse Model of Posttraumatic Stress Disorder

**DOI:** 10.3389/fnins.2022.828382

**Published:** 2022-03-11

**Authors:** Daxue Zhou, Chengyan Long, Yan Shao, Fei Li, Wei Sun, Zihan Zheng, Xiaoyang Wang, Yiwei Huang, Feng Pan, Gang Chen, Yanlei Guo, Yi Huang

**Affiliations:** ^1^Biomedical Analysis Center, Army Medical University, Chongqing, China; ^2^Chongqing Academy of Chinese Materia Medica, Chongqing, China; ^3^Key Laboratory of Extreme Environmental Medicine, Ministry of Education of China, Chongqing, China; ^4^Chongqing Key Laboratory of Cytomics, Chongqing, China

**Keywords:** posttraumatic stress disorder (PTSD), PTSD model, urine, metabolomics, proteomics, mice

## Abstract

Posttraumatic stress disorder (PTSD) is a serious stress disorder that occurs in individuals who have experienced major traumatic events. The underlying pathological mechanisms of PTSD are complex, and the related predisposing factors are still not fully understood. In this study, label-free quantitative proteomics and untargeted metabolomics were used to comprehensively characterize changes in a PTSD mice model. Differential expression analysis showed that 12 metabolites and 27 proteins were significantly differentially expressed between the two groups. Bioinformatics analysis revealed that the differentiated proteins were mostly enriched in: small molecule binding, transporter activity, extracellular region, extracellular space, endopeptidase activity, zymogen activation, hydrolase activity, proteolysis, peptidase activity, sodium channel regulator activity. The differentially expressed metabolites were mainly enriched in Pyrimidine metabolism, D-Glutamine and D-glutamate metabolism, Alanine, aspartate and glutamate metabolism, Arginine biosynthesis, Glutathione metabolism, Arginine, and proline metabolism. These results expand the existing understanding of the molecular basis of the pathogenesis and progression of PTSD, and also suggest a new direction for potential therapeutic targets of PTSD. Therefore, the combination of urine proteomics and metabolomics explores a new approach for the study of the underlying pathological mechanisms of PTSD.

## Introduction

Post-Traumatic Stress Disorder (PTSD) is a persistent stress disorder type that may be delayed or imminent following major psychological trauma (Kessler et al., 1995; Breslau et al., 1998). PTSD can be caused by a variety of major events, including diseases (Kangas et al., 2005; Bush, 2010), war incidents (Owens et al., 2005), natural disasters (Wu et al., 2009), etc. PTSD has four core symptoms according to the Diagnostic and Statistical Manual of Mental Disorders (DSM-5); the re-experiencing of traumatic event(s), continuous avoidance of trauma-related stimuli, negative emotions related to cognitive trauma, and continued increase in alertness (Mahan and Ressler, 2012; Tandon, 2014; Tanaka et al., 2019). Several of these aspects can be captured using situational reminder programming in animal models, leading it to become a common model for studying the symptoms and mechanisms of PTSD. However, the precise molecular changes occurring in these models remains incompletely understood.

Assessments for compositional changes in urine have been demonstrated to have considerable potential for monitoring bodily health (Gao, 2013; Nicholas, 2020). In comparison with blood, urine has the advantages of being non-invasive, convenient to sample repeatedly, biochemical stability, and so on (Wu and Gao, 2015; Jing and Gao, 2018). In addition, urine may not be as strongly regulated by homeostatic mechanisms (Wang et al., 2014; Huang and Lo, 2018). The detection of blood biomarkers usually reflects the relatively stable state in the middle and late stages of the disease (Li, 2015), but misses the signals of short-term changes in the early stage of the disease. In opposite urine, as a blood filter, will collect all the body’s metabolites, thereby detecting more differentiated factors (Li et al., 2014; Gao, 2015). Moreover, recent reports have shown that urine can provide a lot of non-urogenital information, including regarding neuropsychiatric disorders (Emanuele et al., 2010; Marc et al., 2011).

In the past few years, advances in “omics” technology have yielded powerful new tools for biomarker screening, disease mechanism identification, and diagnostic modeling (Petricoin et al., 2006). Cutting-edge “omics” technology has already been deployed to study PTSD. Diverse epigenetic phenomena have enabled researchers to discover conserved molecular mechanisms involved in chromatin modification (Goldberg et al., 2007), especially non-coding RNAs, which play an important role in multiple epigenetic phenomena (Bernstein and Allis, 2005). Studies on PTSD and miRNA have revealed several key contributors to the underlying pathophysiological basis of PTSD (Wingo et al., 2015; Bam et al., 2016a,b; Martin et al., 2017). Genomics research can be used to analyze DNA and RNA sequences by second-generation sequencing and third-generation sequencing techniques to discover new transcripts or exon single nucleotide polymorphisms (SNPS) (Girgenti and Duman, 2018). However, researchers thus far have primarily used blood and postmortem brain tissue to identify biomarkers for PTSD (Thomson et al., 2014; Breen et al., 2015; Bharadwaj et al., 2018). Glycomics studies can analyze the biological functions of all glycans by studying the unique pond group of organisms (Miura and Endo, 2016). Compared with genome sequence discovery, glycomics can better reflect the biological state of complex diseases (Zoldos et al., 2013; Lauc et al., 2016). It has been reported that there are significant changes in the N-glycomic group in psychiatric and neurodegenerative diseases (Vanhooren et al., 2010; Lundstrom et al., 2014; Park et al., 2018). Proteomics studies the complete set of proteins in a biological system (cell, tissue or organism) in a given state at a given time, analyzing changes in protein expression, post-translational modifications, and protein-protein interactions (Wilkins et al., 1996; Jensen, 2006). It is more complex than genomics, but can reflect the precise functional characteristics of proteins (Baloyianni and Tsangaris, 2009). Metabolomics mainly analyses final or intermediate small molecule metabolites produced by gene regulation and can evaluate metabolites altered by treatment or disease (Kaddurah-Daouk and Krishnan, 2009; Mastrangelo et al., 2016). It is reported that metabolomics plays an important role in analyzing the metabolic profile, inflammatory mechanisms and biomarker identification of PTSD (De Bellis et al., 2000; Pace and Heim, 2011; Karabatsiakis et al., 2015; Bersani et al., 2016; de Vries et al., 2016; Hemmings et al., 2017; Mellon et al., 2018; Nedic Erjavec et al., 2018). Therefore, Omics technologies can be further improved study the underlined mechanisms of PTSD and identify diagnostic and prognostic biosignatures.

Despite the promising features of urine biomarkers, the biological interpretation of single typology data is very challenging due to the complexity of urine samples. Therefore, in this study, the analytical capabilities of proteomics and metabolomics were combined to obtain more comprehensive data on mice in the normal group and PTSD group, aiming to discover new potential biomarkers.

## Materials and Methods

### Induction of Electric Foot Shock Stress

**FIGURE 1:**
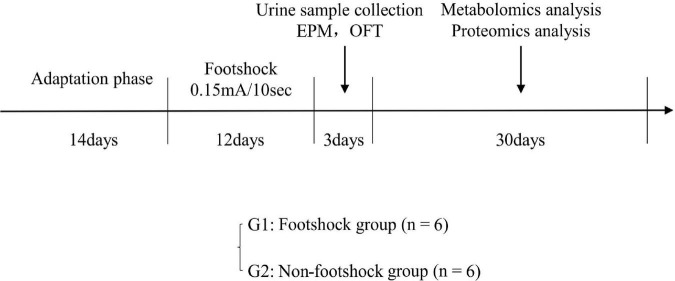
The experimental procedure for PTSD. EPM, elevated plus maze; OFT, open field test.

Twelve healthy male 8–10 weeks old C57BL/6 mice were purchased from Laboratory Animal Centre at the Army Medical University. All mice were housed in individual cages under a reversed 12 h light/12 h dark cycle (light on at 6 AM) and standard laboratory conditions (21 ± 1°C, 55 ± 5% relative humidity). Food and water were provided *ad libitum*. This study was approved by the Ethics Committee of Army Medical University (Animal Ethics Statement: AMUWEC20211605). As shown in Figure 1, after a 14-day adaptation phase, the mice were divided into plantar foot shock group (PTSD group, *n* = 6) and non-foot shock group (control group, *n* = 6). Mice were subjected to electric foot shock in a Plexiglas chamber (27 × 20 × 300 cm3) with a grid floor made of stainless-steel rods (0.3 cm diameter, spaced 1.0 cm apart) connected to a shock generator. After a habituation period of 2 min, the mice in the foot shock group received a series of foot shocks of medium (0.15 mA) intensity of 10 s duration with foot shock interval of 10 s being delivered for 5 min to produce acute stress (Rabasa et al., 2011). The mice in the control group were placed in the chamber for a similar period without receiving a foot shock. Thereafter, the mice in the foot shock group were subjected to the same moderate electric foot shock stressor for 12 days (twice a day) to induce stress adaptation (Van den Berg et al., 1998; Daniels et al., 2008).

### Behavioral Test

All the behavioral tests were performed in daytime from 8 AM to 3 PM. Mice were given two tests a day to avoid the potential interference from the other tests. Animal cages were moved to a testing room at least 0.5 h before each test. After completion of the test session, the behavioral apparatus and chamber were cleaned with 70% ethanol and then completely hand-fan dried.

### Elevated Plus Maze Test

The apparatus consisted of four arms (28 cm × 5.8 cm width), with two arms open and two closed by gray walls (15.5 cm height) arranged on the opposite side of the same type. The platform was located 55 cm above the floor of the testing room illuminated and four arms were connected in the center platform (5 cm × 5 cm), where the animal was placed facing a closed arm. The position and movement of the animal were monitored for 5 min by a video camera. An entry was defined as more than half of the animal’s full body entering the open arm. The time spent in the open arms and the number of visits to the open arms were analyzed.

### Open Field Test

The periphery and bottom of the test space were made of black opaque metal sheets, with length 72 cm, width 72 cm, and height 60 cm. The floor area was divided into 16 squares of the same size. During the test, each mouse was placed in the center of the area and was allowed to freely explore the area for 5 min. The numbers of crossing and standing were recorded during the last 4 min by technicians. After each test, the open area was washed with 70% ethanol to avoid any olfactory cues.

### Urine Sample Collection

Urine was collected on ice using metabolic cages at the end of the experiment from 9:00 pm to 9:00 am in the next day. The collected urine was centrifuged at 13,000 *g* for 20 min at 4°C to obtain the supernatant sample. The average sample size was 2 mL. The urine sample was stored at −80°C before analysis.

### Metabolomics Analysis

Urine samples (50 μL) were thawed on ice and immediately mixed with 200 μL of ice-cold acetonitrile. After mixing by vortex for 1 min, the mixture was centrifuged at 13,000 *g* for 15 min at 4°C. A supernatant aliquot of 10 μL was used for liquid chromatography-mass spectrometry (LC-MS/MS) analysis. Quality control (QC) samples were prepared by supernatant aliquot with an equal amount (15 μL) and were periodically analyzed throughout the complete run to monitor signal drift.

The LC 30A UHPLC system (Shimadzu, Kyoto, Japan) was linked to a Triple TOF 4600 system (SCIEX, Framingham, MA, United States). The separation step was conducted using the hydrophilic interaction liquid chromatography (HILIC) and the reversed-phase liquid chromatography (RPLC) methods. A Kinetex C_18_ column (2.1 mm × 100 mm, 2.6 μm, 100 Å, Phenomenex) was used with a binary gradient method. Solvent A was 0.1% formic acid in water (vol/vol), and solvent B was 0.1% formic acid in acetonitrile (vol/vol). A flow rate of 0.35 mL/min was used, and the injection volume was 2 μL. The gradient program used was 15% B at 0 min to 85% B at 10 min, with a total running time of 15 min. A TSK gel NH_2_-100 column (2.1 mm × 100 mm, 3.0 μm, TOSOH) was also used with a binary gradient method. Solvent A was 5 mmol/L ammonium acetate, and solvent B was acetonitrile. A flow rate of 0.25 mL/min was used, and the injection volume was 2 μL. The gradient program used was 100% B at 2 min to 15% B at 15 min, and at 20 min to 100% B, with a total running time of 25 min.

### Proteomics Analysis

1 mL urine sample was thawed and transferred to a centrifuge tube, and then centrifuged at 12,000 *g* at 4°C for 30 min to remove impurities. The samples were six times mixed with the volume of acetone, fully mixed, and precipitated overnight at – 20°C. The mixture was removed and centrifuged at 12,000 *g* at 4°C for 30 min to remove the supernatant. The precipitate was dissolved in pyrolysis buffer solution (8 mol/L urea, 2 mol/L Thiourea, 50 mmol/L Tris, and 25 mmol/L DTT), and completely dissolved, centrifuged at 12,000 *g* for 30 min at 4°C, and then the supernatant was saved. Protein concentration was determined using the Bradford method. 100 ug protein was added to each sample in a 30 KDa filter (millipore, MRCF0R030), Urea buffer solution (UA, 8 mol/L, 0.1 mol/L Tris–HCl, pH 8.5), and 25 mmol/L NH_4_HCO_3_ solutions were in turn washed several times. Protein samples were reduced with 20 mmol/L dithiothreitol (DTT, Sigma) at 37°C for 1 h, followed by 50 mmol/L iodoacetamide (IAA, Sigma) in darkness for 30 min. Then, the samples were centrifuged at 18°C for 30 min at 14,000 *g*, washed with UA and NH_4_HCO_3_, with trypsin being added (enzyme protein ratio 1:50) and digested overnight at 37°C. The peptide mixture was desalted using a C_18_ column (Thermo, 84850), concentrated, dried in vacuum, and stored at −80°C.

AU3000 UHPLC system (Thermo Fisher Scientific, Waltham, MA, United States) was used to separate the peptides. Peptides were loaded onto an analytical column (Acclaim™ PepMap™ 100, 75 μm × 15 cm, C_18_, 3 μm, 100 Å, Thermo Fisher Scientific, Waltham, MA, United States) with a Trap Column (Acclaim™ PepMap™ 100, 75 μm × 2 cm, C18, 3 μm, 100 Å, Thermo Fisher Scientific, Waltham, MA, United States) and separated by reversed-phase chromatography (U3000nano, Thermo Fisher Scientific, Waltham, MA, United States) using a 106 min gradient. The gradient was composed of Solvent A (0.1% formic acid in water) and Solvent B (0.1% formic acid in 80% acetonitrile) elution gradient: 1% B for 13 min, 1–30% B in 70 min, 30–90% B in 10 min, 90% B for 2 min and 90–1% B in 1 min, 1% B for 10 min. The eluted peptides were analyzed using the Data Dependent Acquisition (DDA) method applying one full MS scan (350.00–1800.00 m/z) in the Orbitrap at a resolution of 60,000 M/ΔM, followed by consecutive MS/MS (profile) scans in the ion trap by product ion scans (relative CID energy 35) of the 16 most abundant ions in each survey scan. The product ion scans were acquired with a 2.0 unit isolation width and a normalized collision energy of 35 in an LTQ-Orbitrap Velos Pro MS spectrometer (Thermo Fisher Scientific, Waltham, MA, United States).

### Statistical Analysis

Statistical analyses were performed using SPSS 20.0 software, values are expressed as mean ± standard deviation (X ± SD, *n* = 6 per group), the graphics were generated using GraphPad Prism 8.0.1 software. Metabolomics and Proteomics data analyses were performed in MetaboAnalyst 5.0 and Proteome Discover Daemon 2.5. The metabolite peaks of the urine samples were normalized, analyses performed using SIMCA-P 14.1 multivariate statistical analysis software. All variables were tested and found to be normally distributed, an independent-samples student’s *t*-test was used to compare differences between the two groups, and identify differentially expressed metabolites and proteins, and then we used Ingenuity Pathway Analysis to analyze the significantly altered canonical pathways and molecular interaction networks. A *p*-value threshold of 0.05 was used to infer statistically significant findings, and a more strict *p*-value threshold of 0.01 was used to infer highly statistically significant changes.

## Results

### The Results of the Behavioral Test

#### Elevated Plus Maze Test

**FIGURE 2:**
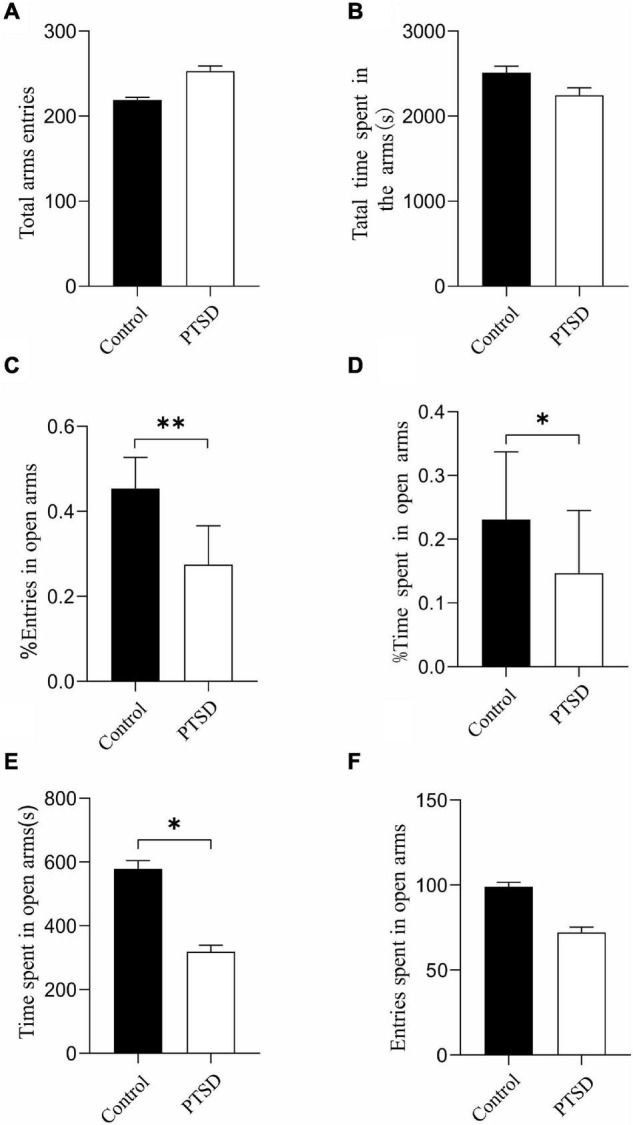
The effect of the induction of electric foot shock on anxiety in the elevated plus-maze test in mice compared to the control group (*n* = 6). **(A)** Total arms entries; **(B)** Total time spent in the arms; **(C)** The percent of entries in open arms; **(D)** The percent of time spent in open arms; **(E)** The actual times spent in open arms; **(F)** The actual amount of entries in open arms. Results are provided in the form of mean ± SD. *p < 0.05; **p < 0.01.

Elevated plus maze test was deployed to explore the potential anxiety actions of the induction of electric foot shock stress. There was no significant difference in terms of total arms entries (Figure 2A) and total time spent in the arms (Figure 2B) between the control and the PTSD groups.

The induction of the electric foot shock stress caused a significant reduction in the percent of open arm entries (open arm/total × 100) with the ones of the control and the PTSD group being 45.4 and 27.5%, respectively (Figure 2C). The percent of time spent in open arms was also significantly reduced when applying induction of electric foot shock stress with control and PTSD group values being 23.1 and 14.7%, respectively (Figure 2D). The actual times spent in open arms in the control group and the PTSD group were 578 s and 319 s, respectively (Figure 2E). The actual amount of entries in open arms in the control group and the PTSD group were 99 and 72, respectively (Figure 2F).

#### Open Field Test

**FIGURE 3:**
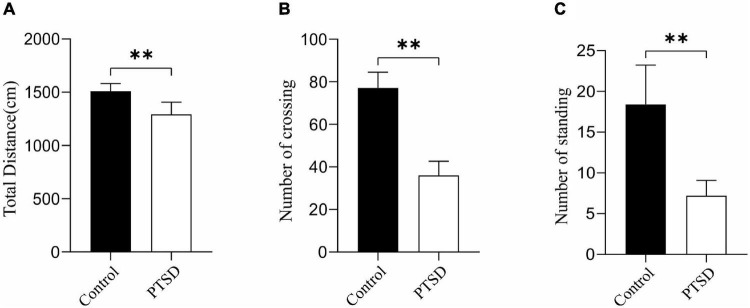
The effect of the induction of electric foot shock on anxiety in the open-field test in mice compared to the control group (*n* = 6). **(A)** Overall distance; **(B)** Numbers of crossing; **(C)** Numbers of standing. Data are expressed as mean ± SD. **p < 0.01.

The anxiety-like behavior of the induction of electric foot shock stress was measured with an open-field test. The overall distance was significantly reduced when applying induction of electric foot shock stress with the values of the control and PTSD groups being 1511.18 and 1292.94 cm, respectively (Figure 3A). The number of crossing and standing was also significantly reduced by the Induction of electric foot shock stress. The numbers of crossing in the control group and the PTSD group were 77.1 and 36.0 times, respectively (Figure 3B). The amount of times of standing in the control group and the PTSD group were 18.4 and 7.2 times, respectively (Figure 3C).

### Metabolomics Analysis

**FIGURE 4:**
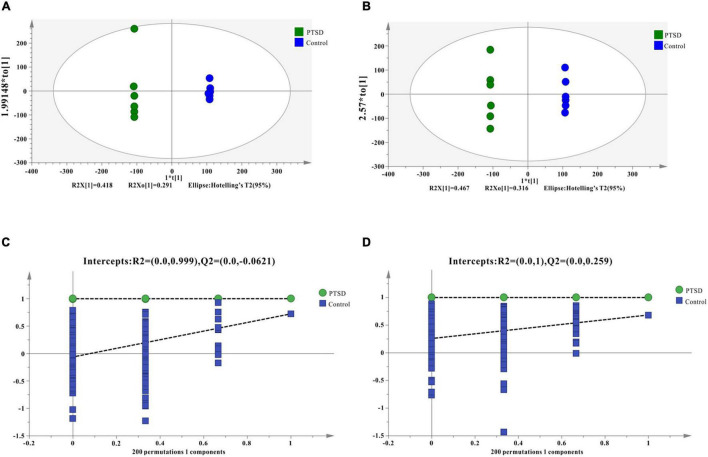
OPLS-DA score results and OPLS-DA quality control figure of mice urine samples. **(A)** Positive ion mode OPLS-DA scores; **(B)** Negative ion mode OPLS-DA scores; **(C)** Positive ion mode OPLS-DA permutation test; **(D)** Negative ion mode OPLS-DA permutation test; intercepts: R2 and Q2 represent y-intercept of R2 and Q2 regression lines.

Quality control results pinpointed that the variation caused by instrument error is small and the data quality is reliable (Supplementary Figure 1). The PCA results plot does not show clear segregation between the PTSD group and the control group (Supplementary Figure 2). In addition, The OPLS-DA model was established and a permutation test of the OPLS-DA model was performed (positive mode: R2X = 0.919, R2Y = 1.0, Q2 = 0.723; negative mode: R2X = 0.854, R2Y = 0.998, Q2 = 0.657; Figures 4A,B). The results of the permutation test showed the absence of overfitting (positive mode: R2 = 0.999, Q2 = −0.0431; negative mode: R2 = 0.995, Q2 = −0.0933; Figures 4C,D). In conclusion, the model presented good reliability and predictability.

### Differential Metabolites

**FIGURE 5:**
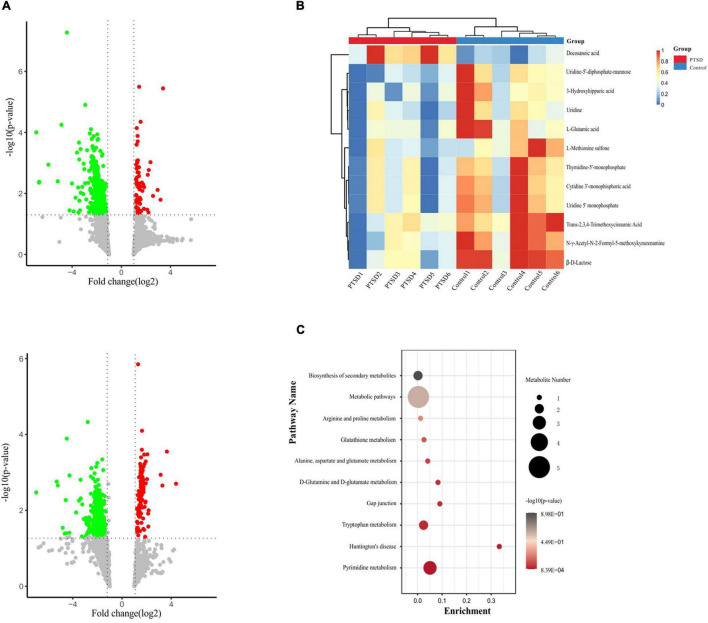
Metabolites profile shift during of mice urine samples between the PTSD group and the control group. **(A)** Volcano plot showing altered metabolites, Positive (up), negative (down) ion mode. The red dots indicate significantly upregulated metabolites (fold change > 1.2), while the green dots indicate significantly downregulated metabolites (fold change < 0.8). **(B)** Heat map of the differentially expressed metabolites. The red band indicates the upregulation of metabolites levels (fold change > 1.2), while the blue band indicates the downregulation of metabolites levels (fold change < 0.8). **(C)** KEGG pathway enrichment category of the differentially expressed metabolites, *n* = 6.

Metabolomics profiling of urine from the C57BL/6 normal group mice and the PTSD mouse-model group detected a total of 559 metabolite components and revealed 12 differentially expressed metabolites between the PTSD group and the Control group using as criteria to infer significant findings the VIP > 1 and *p* < 0.05 (Figure 5A and Table 1). These differentially expressed metabolites possess different characteristics (Figure 5B) and were enriched for several KEGG pathways associated with amino acid and nucleic acid metabolism, including Pyrimidine metabolism, D-Glutamine and D-glutamate metabolism, Alanine, aspartate and glutamate metabolism, Arginine biosynthesis, Glutathione metabolism, Arginine, and proline metabolism (Figure 5C).

**TABLE 1:** The significantly differentiated metabolites in control vs. post-traumatic stress disorder (PTSD) groups.

No	Metabolites	VIP	FC	*P*-value	Trend
1	L-Methionine sulfone	1.27643	0.39592211	0.00128436	↓
2	Docosanoic acid	1.11139	1.24292539	0.00153343	↑
3	Uridine	2.21959	0.54969523	0.00295338	↓
4	3-Hydroxyhippuric acid	1.10166	0.58905271	0.00886306	↓
5	L-Glutamic acid	1.04933	0.42636368	0.00901154	↓
6	Uridine 5′ monophosphate(UMP)	2.42806	0.47128583	0.02161141	↓
7	*Trans*-2,3,4-Trimethoxycinnamic Acid	1.05031	0.46980814	0.02427128	↓
8	β-D-Lactose	1.65882	0.51933915	0.02631537	↓
9	Cytidine 3′-monophisphoric acid	3.48481	0.49014935	0.02692515	↓
10	N-γ-Acetyl-N-2-Formyl-5-methoxykynurenamine	1.5343	0.52282757	0.02905089	↓
11	Thymidine-5′-monophosphate(dTMP)	7.26583	0.50788472	0.03078474	↓
12	Uridine-5′-diphosphate-mannose(UDP-Gal)	1.16583	0.49116102	0.03410945	↓

### Proteomics Analysis

**FIGURE 6:**
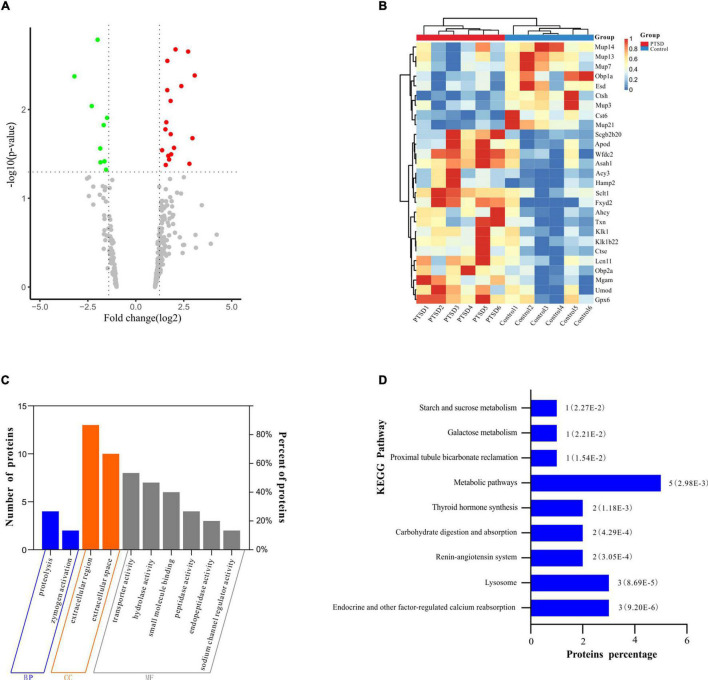
Proteomic profile shift of mice urine samples between the PTSD group and the control group. **(A)** Volcano plot showing dysregulated proteins. The red dots indicate significantly upregulated proteins (fold change > 1.2), while the green dots indicate significantly downregulated proteins (fold change < 0.8); **(B)** Heat map of the differentially expressed proteins. The red band indicates the upregulated proteins (fold change > 1.2), while the blue band indicates the downregulated proteins (fold change < 0.8); **(C)** Differentially expressed protein GO function enrichment diagram; **(D)** KEGG pathway enrichment of the differentially expressed proteins, *n* = 6.

The number of peptide-spectral matches, unique peptide number, and quantified proteins, were 88,734, 4,125, and 691 for both PTSD and control groups. 27 proteins exhibited significantly differentiated expression between the two groups using the criteria of *p*-value < 0.05 and fold change > 1.20 or <0.80. A total of 18 proteins among these were upregulated and 9 downregulated in the PTSD group compared to the Control group (Figure 6A and Table 2). These altered features were subjected to clustering, and the heat map revealed clusters with the ability to discriminate between control and PTSD samples (Figure 6B). Gene Ontology (GO) function annotation analysis showed that these differentially expressed proteins were mainly involved in biological processes, such as small molecule binding, transporter activity, extracellular region, extracellular space, endopeptidase activity, zymogen activation, hydrolase activity, proteolysis, peptidase activity and sodium channel regulator activity (Figure 6C). Based on the KEGG database, the significantly enriched pathways (*P* < 0.05) were Endocrine and other factor-regulated calcium reabsorption, Lysosome, Renin-angiotensin system, Carbohydrate digestion and absorption, Thyroid hormone synthesis, Metabolic pathways, Proximal tubule bicarbonate reclamation, Galactose metabolism and Starch and sucrose metabolism (Figure 6D).

**TABLE 2:** The differentially expressed proteins in control vs. PTSD groups.

No	UniProt accession	Gene symbol	Protein name	FC	*P*-value	Trend
1	P49935	Ctsh	Pro-cathepsin H	−1.99023949	0.00163517	↓
2	G5E861	Sclt1	Sodium channel and clathrin linker 1	2.07219424	0.00209911	↑
3	A0A1L1SQP8	Fxyd2	FXYD domain-containing ion transport regulator	2.72899236	0.00222118	↑
4	Q3TWT5	Asah1	Ceramidase	1.64659341	0.00282754	↑
5	J3QK77	Scgb2b20	ABPBG20	3.07210788	0.00413095	↑
6	Q9D1B1	Cst6	Cystatin E/M	−3.19900397	0.00422517	↓
7	A0A571BF69	Mgam	Maltase-glucoamylase	2.37524214	0.00543735	↑
8	Q4FZJ6	Wfdc2	WAP four-disulfide core domain 2	1.64128707	0.00604615	↑
9	Q8K1H9	Obp2a	Odorant-binding protein 2a	1.81533979	0.00799517	↑
10	Q80YX8	Mup21	Major urinary protein 21	−2.29692495	0.00913460	↓
11	Q3KQQ2	Mup3	Major urinary protein 25	−1.49751001	0.01240960	↓
12	Q91WR8	Gpx6	Glutathione peroxidase 6	1.59143645	0.01390856	↑
13	Q9D3H2	Obp1a	Odorant-binding protein 1a	−1.67221913	0.01499012	↓
14	A2BHR2	Lcn11	Lipocalin 11	1.54440618	0.01672011	↑
15	Q3TF14	Ahcy	Adenosylhomocysteinase	1.82072680	0.01895520	↑
16	A0A0U1RPF4	Hamp2	Hepcidin-2 (Fragment)	2.95442297	0.02102362	↑
17	P10639	Txn	Thioredoxin	1.993343846	0.027021861	↑
18	A2CEK6	Mup13	Major urinary protein 11	−1.85239538	0.02745480	↓
19	P15947	Klk1	Kallikrein-1	1.37162847	0.02874193	↑
20	P70269	Ctse	Cathepsin E	1.84456216	0.03194560	↑
21	P51910	Apod	Apolipoprotein D	1.69316293	0.03324157	↑
22	P15948	Klk1b22	Kallikrein 1-related peptidase b22	1.73666194	0.03657697	↑
23	H3BKH6	Esd	S-formylglutathione hydrolase	−1.63242181	0.03836365	↓
24	L7MUC7	Mup7	Major urinary protein 7 (Fragment)	−1.84000481	0.03930886	↓
25	Q91XE4	Acy3	N-acyl-aromatic-L-amino acid amidohydrolase (carboxylate-forming)	2.79646484	0.04085258	↑
26	Q91 × 17	Umod	Uromodulin	1.56345644	0.04212900	↑
27	B8JI96	Mup14	Major urinary protein 14 (Fragment)	−1.54699463	0.04765555	↓

### Integrative Analysis of the Metabolomics and Proteomics

**FIGURE 7:**
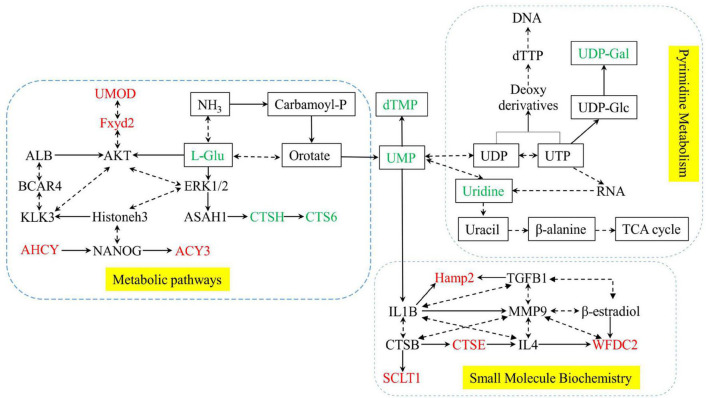
Network of significantly differential metabolic pathways for Posttraumatic stress disorder. Yellow dashed areas represent the pathways. metabolites are shown as rectangles, and Proteins are shown without no rectangles. Red represents significant upregulation in the PTSD group compared to the control group, green represents significant downregulation.

A total of 12 differential expression metabolites and 27 differential expression proteins that were submitted to Ingenuity Pathway Analysis (IPA) for significantly altered canonical pathways analysis. As shown in Table 3, We found three pathways significantly expressed proteins and metabolites. They were Pyrimidine Metabolism, Metabolic pathways, and Small Molecule Biochemistry. These significantly differential metabolic pathways were selected for more detailed analysis (Figure 7). In these pathways, L-Glutamic acid(L-Glu), Uridine5-monophosphate(UMP), Thymidine 5-monophosphate(dTMP), Uridine, URIDINE-5′-DIPHOSPHATE-MANNOSE(UDP-Gal), CTSH and CTS6 were downregulated, and UMOD, Fxyd2, AHCY, ACY3, Hamp2, CTSE, SCLT1, WFDC2 were upregulated.

**TABLE 3:** Significantly altered pathways with differentially expressed proteins and metabolites.

No	Pathway name	Proteins	Metabolites
1	Pyrimidine metabolism		Uridine, UMP, dTMP, UDP-Gal
2	Metabolic pathways	UMOD,Fxyd2,AHCY,ACY3	L-Glu
3	Small Molecule Biochemistry	Hamp2,CTSE,SCLT1,WFDC2	UMP

## Discussion

Currently, diagnosis of PTSD is primarily based on subjective symptom representation and patient self-reporting, and the molecular mechanism remains unclear. As such, rates of PTSD in the general population may be significantly underestimated. In the present manuscript, we established a mouse model of PTSD to investigate some of its qualitative biomarkers and potential mechanistic contributors. The elevated cross maze and open-field test were evaluated based on the fact that the plantar shock can continuously produce traumatic stimulation. The mice with the PTSD group were observed to have reduced movement, weakened active exploration ability, and showed negative avoidance and anxiety in comparison to the mice of the control group (Montgomery, 1955; Pellow et al., 1985), indicating significant stress disorder. Combined proteomics and metabolomics analysis was performed revealing 27 significantly dysregulated proteins and 12 significantly dysregulated metabolites.

In this study, urinary uridine levels in the mice model were significantly reduced, suggesting that PTSD can cause metabolic abnormalities of uridine in urine. It has been reported that uridine has a protective effect on mental disorders (Mironova et al., 2018) and can improve neurophysiological functions (Connolly and Duley, 1999). Uridine excretion is mainly achieved through renal and pyrimidine metabolism, producing uracil and β -alanine, which can enter the tricarboxylic acid (TCA) cycle (Gonzalez and Fernandez-Salguero, 1995; Connolly et al., 1996). The homeostasis and metabolic abnormalities of uridine can be accurately monitored by the detection of uridine in urine.

In mammals, in pyrimidine metabolism, uridine (UR) is involved in the de initio synthesis of uridine monophosphate (UMP) to form uridine 5′ -diphosphate (UDP), which can be combined with UDP-galactose and plays an important role in the glycosylation of protein (Connolly and Duley, 1999). It has been reported that pyrimidines are mainly recovered from uridine, which synthesizes RNA and biofilms through pyrimidine nucleotide – lipid conjugates (Yamamoto et al., 2011). In the study, the urine metabolism of uridine (UR), uridine monophosphate (UMP), and UDP-galactose in mice of the PTSD model group showed decreased expression, suggesting that the PTSD mice induced by plantar electric shock exhibit disorder of pyrimidine metabolism.

Glutamate (L-glutamate) is a major excitatory neurotransmitter, and glutamate disorder in the brain is often observed in depression models (Hemanth Kumar et al., 2012; Liu et al., 2016). In this study, the expression of L-glutamate in urine metabolism was decreased in the PTSD model group, while glutamate can provide a nitrogen source for pyrimidine synthesis (Vincenzetti et al., 2016). The pyrimidine metabolism disorder can directly reflect abnormal glutamate metabolism. Studies have reported that patients with PTSD and alcohol use disorder (AUD) have significantly reduced glutamate in the anterior cingulate cortex (ACC; Pennington et al., 2014). Glutamate is the basis of synaptic plasticity and memory formation, and stress response significantly affects glutamate transmission and plays a key role in PTSD (Chambers et al., 1999; Lamprecht and LeDoux, 2004; Popoli et al., 2011; Kelmendi et al., 2016). Urine collects all metabolites of the body and is not regulated by the homeostasis mechanism. Abnormal l-glutamate metabolism detected in urine directly reflects PTSD.

In addition to metabolomic changes, significant proteomics differences were also identified. GO analysis of the urine proteome data showed that proteins with differential expression were mainly located in the extracellular space and extracellular region. It mainly binds to small-molecule, and it is involved in hydrolase activity, endopeptidase activity, and sodium channel regulator activity. Pathway enrichment analysis showed that these proteins are mainly involved in Endocrine and other factor-regulated calcium reabsorption, Lysosome, Renin-angiotensin system, Carbohydrate digestion and absorption, Metabolic pathways, etc.

Hepcidin is a circulating antimicrobial peptide involved in iron homeostasis, inflammation, infection, and metabolic signaling (Lu et al., 2015), There are two murine hepcidin genes: hepcidin-1 (Hamp1) and hepcidin-2 (Hamp2) (Truksa et al., 2007). Studies have shown that in addition to liver level, inflammation can increase the expression level of iron modulin (Kanamori et al., 2017; Silva et al., 2019). In this study, hepcidin-2 (Hamp2) expression increased. It has been reported that people with PTSD show elevated levels of pro-inflammatory cytokines, including IL1B (Dinarello, 2011; Tursich et al., 2014; Passos et al., 2015). In animal studies, IL1B expression level in hippocampus of depressed animal model was increased (Goshen et al., 2008). Therefore, Hamp2 expression may be induced by inflammatory factors in mice with PTSD. At the same time, IPA analysis showed that increased Hamp2 expression was correlated with IL1B, and the relationship between Hamp2 and IL1B in THE urine of PTSD will be further discussed in subsequent studies.

There are some limitations in the present study. First, the abundance of metabolites and proteins in the urine itself is small, and removing the peak degree through database construction ends up in data-loss, suggesting a potential data loss in urine protein-metabolism combined analysis. Second, only 6 eligible mice from each group were used for protein-metabolic analysis, and the results of the discovery omics study were not validated by targeted methods (e.g., western blotting). Therefore, further studies are required to validate these findings.

## Conclusion

In this study, based on urine protein-metabolomics combined analysis, we found that the differentially expressed proteins of PTSD in mice were mainly in the extracellular space and region, and showed dysfunction of pyrimidine metabolism. Furthermore, Uridine and L-glutamate can be used as key urine biomarkers to provide a reference for subsequent studies on PTSD.

## Data Availability Statement

The raw data supporting the conclusions of this article will be made available by the authors, without undue reservation.

## Ethics Statement

The animal study was reviewed and approved by the Ethics Committee of Army Medical University.

## Author Contributions

DZ, YH, and FL contributed to the design. XW, YWH, WS, and CL participated in the animal experiments. FP, YG, ZZ, and YS assisted to analyze the data. DZ drafted and modified the manuscript. YH and GC provide to financial support. All authors have read and agreed to the manuscript for submission.

## Conflict of Interest

The authors declare that the research was conducted in the absence of any commercial or financial relationships that could be construed as a potential conflict of interest.

## Publisher’s Note

All claims expressed in this article are solely those of the authors and do not necessarily represent those of their affiliated organizations, or those of the publisher, the editors and the reviewers. Any product that may be evaluated in this article, or claim that may be made by its manufacturer, is not guaranteed or endorsed by the publisher.
