# Supplementary material for: Integrated Metabolomics and Proteomics Analysis of Urine in a Mouse Model of Posttraumatic Stress Disorder
Source: Front Neurosci. 2022 Mar 11;16:828382. doi: 10.3389/fnins.2022.828382 (PMC8963102; doi:10.3389/fnins.2022.828382)
Supplement: Supplementary file 1 [file Data_Sheet_1.docx]

Supplementary Material

# Supplemental Figure


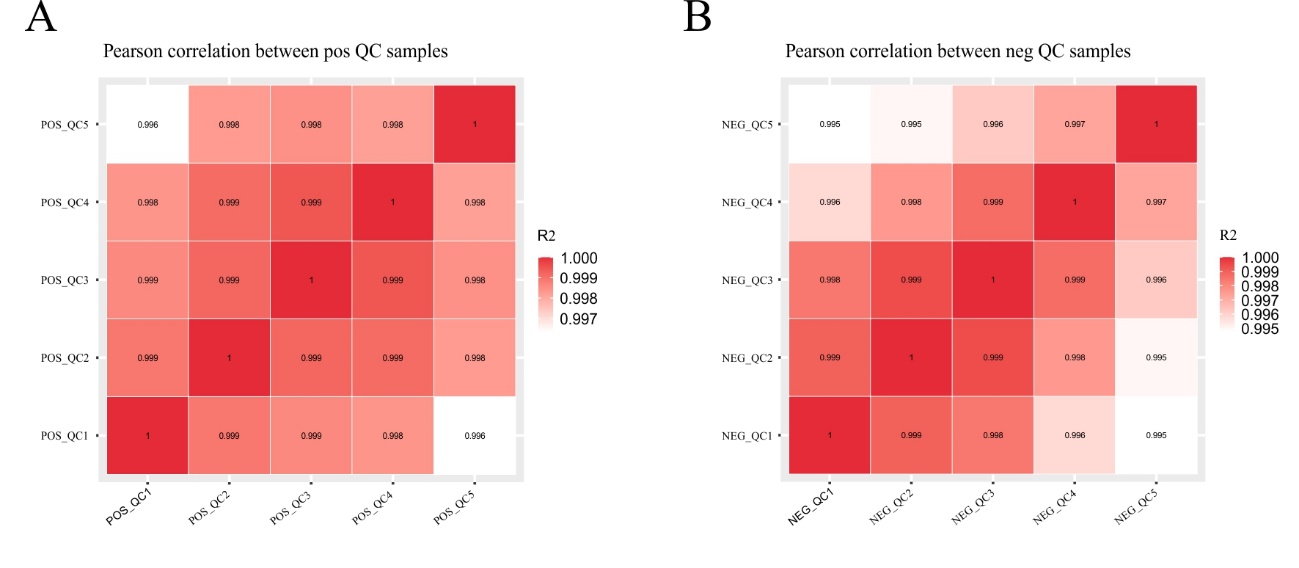


Supplementary Figure 1. The Pearson’s correlation coefficient among QC samples. (A) positive ion mode; (B) Negative ion mode.


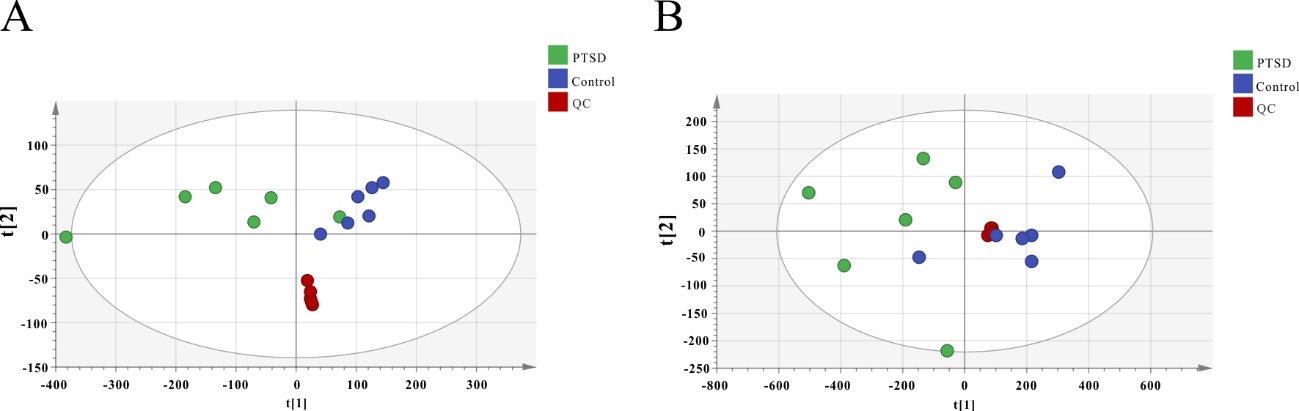


Supplementary Figure 2. PCA Results. (A) positive ion mode; (B) Negative ion mode.
